# Rational Construction of Nano-Scaled FeOOH/NiFe-LDH for Efficient Water Splitting

**DOI:** 10.3390/nano15120949

**Published:** 2025-06-18

**Authors:** Juan Yu, Xiubing Fu, Haoqi Wang, Shun Lu, Bing Li

**Affiliations:** 1 Anhui Province Key Laboratory of Pollutant Sensitive Materials and Environment Remediation, Anhui Province Industrial Generic Technology Research Center for Alumics Materials, School of Physics and Electronic Information, Huaibei Normal University, Huaibei 235000, China; 2 Radiation Technology Institute, Beijing Academy of Science and Technology, Beijing 100875, China; 3 Chongqing Institute of Green and Intelligent Technology, Chinese Academy of Sciences, Chongqing 400714, China

**Keywords:** water splitting, transition metal hydroxides, oxygen evolution reaction, hydrogen evolution reaction, interface engineering

## Abstract

In this paper, we use the facile approach for preparing novel, low-cost, efficient electrocatalysts for electrocatalytic water splitting. Interfacial engineering can significantly enhance the intrinsic performance of electrocatalysts. Herein, self-supporting FeOOH/NiFe-layered double hydroxide (LDH) nanosheet arrays were synthesized via hydrothermal and impregnation methods. The resulting FeOOH/NiFe-LDH can provide more active regions, which provide more active regions for co-reaction to proceed and accelerates electron transmit processes. Additionally, the amorphous FeOOH provides abundant active sites with low coordination, leading to excellent activity. The FeOOH/NiFe-LDH demonstrates remarkable two half-reaction electrocatalytic activity, along with excellent overpotentials of 168 mV (OER) and 155 mV (HER). This research introduces a sophisticated and scalable methodology for the creation of remarkably efficient and resilient alkaline conditions specifically designed for the HER and OER.

## 1. Introduction

While traditional fossil energy sources provide convenient energy, the heavy depletion and consumption of mineral fuels and the consequent ecological issues have seriously affected the living environment [1,2]. As is well known, hydrogen stands out as a promising environmentally friendly energy source, attributed to its distinctive advantages [3,4,5]. The water electrolysis included the hydrogen evolution reaction (HER) and the oxygen evolution reaction (OER) [6,7,8]. However, both half reactions exhibit sluggish reaction kinetics and require efficient electrocatalysts to reduce overpotential and improve reaction efficiency [9,10,11]. Although precious metal catalysts for Pt for the HER and RuO_2_/IrO_2_ for the OER demonstrate excellent performance, the high costs of preparation, poor durability in use, and insufficient durability severely impede widespread industry implementation [12,13]. The quest for affordable and highly effective OER catalysts continues to pose a pressing challenge in the pursuit of alternatives to noble metal materials [14,15,16]. An efficient and abundantly available electrocatalyst for the HER and OER is indispensable for achieving efficient and economically viable electrochemical water splitting [17,18].

Transition metal elements are inherently favored, attributed to their economic efficiency, wide accessibility, and unique physical and chemical attributes [19,20,21]. These metals possess unique orbitals and lower activation energy of the crystal field, which contribute to enhanced catalytic activity. Previous studies have demonstrated that compounds comprising transition metals such as nickel, iron, and cobalt are considered appealing alternatives [11,22]. Numerous electrocatalysts, including chalcogenides, phosphides, borides, and metal oxides/hydroxides, have been investigated. Bimetallic hydroxides are particularly advantageous for catalytic reactions, benefiting from their expansive specific surface area and the interchangeability of interlayer anions [10,23,24]. Among these materials, nickel–iron-layered double hydroxide (NiFe-LDH)—characterized by its brucite-like layered structure with intercalated anions and a tunable Ni^2+^/Fe^3+^ ratio—has garnered extensive research attention due to its exceptional electrocatalytic properties. The theoretical calculation results in a large number of studies prove the key influence of the electron density distribution of metal active sites in LDH materials on catalytic performance and clarify the effect of anion exchange between LDH layers on catalytic stability [25,26,27]. A considerable number of endeavors have been dedicated to improving the electrocatalytic properties of NiFe LDH through modifications of its chemical composition or the engineering of diverse nanostructures. The preparation methods reported for NiFe-based (oxygen) hydroxides face challenges in simultaneously achieving ultralow cost, high performance, and ease of large-scale production [28,29]. For example, Gao et al. used the hydrothermal method to synthesize Ni/NiFe-LDH nanosheets, achieving impressive overpotentials of 150 mV and 205 mV at 10/30 mA cm⁻^2^ during the two half-reactions [30,31]. Fe, a wise selection due to its cost-effectiveness and environmental friendliness, garners particular attention, as even trace quantities of Fe can significantly improve the two half-reactions’ activity of Ni-based (oxy)hydroxide. Furthermore, metal oxyhydroxides function as highly potent electrocatalysts for the HER and OER and are acknowledged as pivotal active phases in the water electrolysis process [32,33,34]. Wang and co-workers successfully prepared FeOOH/Co_9_S_8_ catalysts through s hydrothermal reaction and electrodeposition modification onto nickel foam (NF). The resultant catalyst boasts abundant heterogeneous interfaces, which optimizes the coordination environment of Fe and Co sites. This synergistic effect not only enhances the adsorption of oxygen-containing mesophase but also accelerates the reaction kinetics. Notably, FeOOH/Co_9_S_8_-15 achieves an overpotential of 248 mV at 10 mA cm⁻^2^ and maintains remarkable stability for over 140 h [35,36]. Additionally, under typical electrochemical conditions (especially alkaline OER), these hydroxides and oxides exhibit excellent stability, following the trend FeO_x_H_y_ > CoO_x_H_y_ > NiO_x_H_y_ [37,38,39]. Numerous studies have shown that amorphous materials have the structural characteristics of long-range disorder and short-range order, which makes active sites more accessible on the exterior of the material. In catalytic reaction, the superior structural flexibility of amorphous materials allows the active sites to be transformed into any shape required for the adsorption of active intermediates, thus improving the activity of unit active sites. Amorphous materials have garnered significant interest for their unique structural and electronic properties. FeOOH offers abundant active sites, a significant quantity of surface defects, and low-coordination environments, which collectively contribute to improved HER and OER activity [40,41,42]. Recent advances highlight the potential of heterostructure engineering to address these limitations. Nevertheless, most existing FeOOH/LDH composites rely on crystalline FeOOH phases synthesized via energy-intensive methods, which suffer from insufficient active site exposure and interfacial stress-induced delamination [43,44,45].

Herein, FeOOH/NiFe-LDH was synthesized via the hydrothermal-assisted solution impregnation method. This catalyst exhibited excellent catalytic activity for water electrolysis. Through the distribution of the Ni-to-Fe ingredient, the electronic states of surface atoms were modulated. Furthermore, etching of the LDH nanosheets through interface modification increased the specific surface area, improving reaction efficiency. The FeOOH/NiFe-LDH synthesized through these two modification methods demonstrated robust stability for a 200 h continuous stability test under varying current densities. The FeOOH/NiFe-LDH composite, with an optimized ratio of components, exhibited superior catalytic activity and stability compared to Ni(OH)_2_ and Fe(OH)_3_. FeOOH/NiFe-LDH is characterized by its good stability, low resistivity, and superior intrinsic activity. The decoration of the interface with amorphous iron oxyhydroxide significantly decreased the electrical impedance to charge transfer throughout the water electrolysis catalysis, thereby boosting electrocatalytic performance.

## 2. Experimental Section

### 2.1. Materials

The synthetic process contains nickel chloride hexahydrate (NiCl_2_·6H_2_O), iron (III) chloride hexahydrate (FeCl_3_·6H_2_O), urea (CO(NH_2_)_2_), ammonium fluoride (NH_4_F), ferrous sulfate tetrahydrate (FeSO_4_·H_2_O), potassium hydroxide (KOH), hydrochloric acid (HCl), anhydrous deionized water (DI water), and Ni foam (1.5 mm). (All reagents are Sinopharm Group, nickel foam origin China Kunshan Energy New Materials Co., Ltd., Kunshan, China)

### 2.2. Preparation of Ultrathin Ni-Fe LDH

Various ratios of NiCl_2_·6H_2_O and FeCl_3_·6H_2_O, 1.0 g of urea, and 0.14 g of NH_4_F were dissolved in 25 mL of DI water and stirred for 30 min at ambient temperature. This homogeneous mixture was then transferred to a 50 mL stainless steel autoclave; we put a piece of treated foamed nickel into the reaction kettle for solvothermal reaction at 160 °C for 12 h. Once the reaction temperature descended to the thermal state, the desired product was isolated from the solution, washed thoroughly with deionized water and absolute ethanol, and then dried in a vacuum oven at 60 °C overnight. Subsequently, a range of products were obtained.

### 2.3. Preparation of FeOOH/NiFe-LDH

The Ni-Fe LDH sample was briefly submerged in a 100 mmol/L FeSO_4_ solution for a few minutes, followed by thorough rinsing and multiple centrifugations using deionized water and alcohol. Afterward, it was placed in a vacuum-drying oven for further utilization.

### 2.4. Physical Characterizations

The instrument parameters required for testing the physical and chemical characteristics of the samples are as follows: Powder X-ray diffraction (XRD) patterns were obtained with a PAN analytical Empyrean X-ray diffractometer, employing Cu Kα radiation (λ = 1.5418 Å) within the diffraction angle range of 2θ = 10–80°. Morphological details were characterized by field emission scanning electron microscopy (FESEM, REGULUS-8220, Tokyo, Japan, HITACHI) and transmission electron microscopy (TEM, JEM-2100, Tokyo, Japan, Electronics). High-resolution X-ray photoelectron spectroscopy (XPS, Kratos Axis Supra+, Tokyo, Japan, Shimadzu) was utilized to verify the chemical composition and exterior electronic states.

### 2.5. Electrochemical Measurements

All electrochemical tests were performed by a three-electrode system on the CHI760E electrochemical workstation (Chenhua760e, Shanghai, China). The working electrode is the as-prepared nickel foam (1 cm^2^); the counter electrode and reference electrode are graphite rod and Hg/HgO. The overpotential (η) was calculated using the following equation: η = E_RHE_ − 1.23 V. The electrolyte was 1.0 M KOH solution. The setting parameters of linear sweep voltammograms (LSVs) are at a scan rate of 5 mV s^−1^. The setting parameters of the electrochemical impedance spectra (EIS) measurements are obtained in the frequency range from 100 kHz to 0.01 Hz, corresponding to the voltage at a current density of 10 mA cm^−2^. To calculate the C_dl_, CV measurement parameters were used at diverse scanning velocity. The current densities (Δj = |ja − jc|/2) obtained versus scan rate were plotted, and the slopes of the fitting curves match C_dl_. For all potentials, we referenced to the reversible hydrogen electrode (vs. RHE), E_vs.RHE_ = E_vs.Hg/HgO_ + 0.098V + 0.059 × pH.

## 3. Results and Discussion

### 3.1. Physicochemical Properties of FeOOH/NiFe-LDH

**Figure 1:**
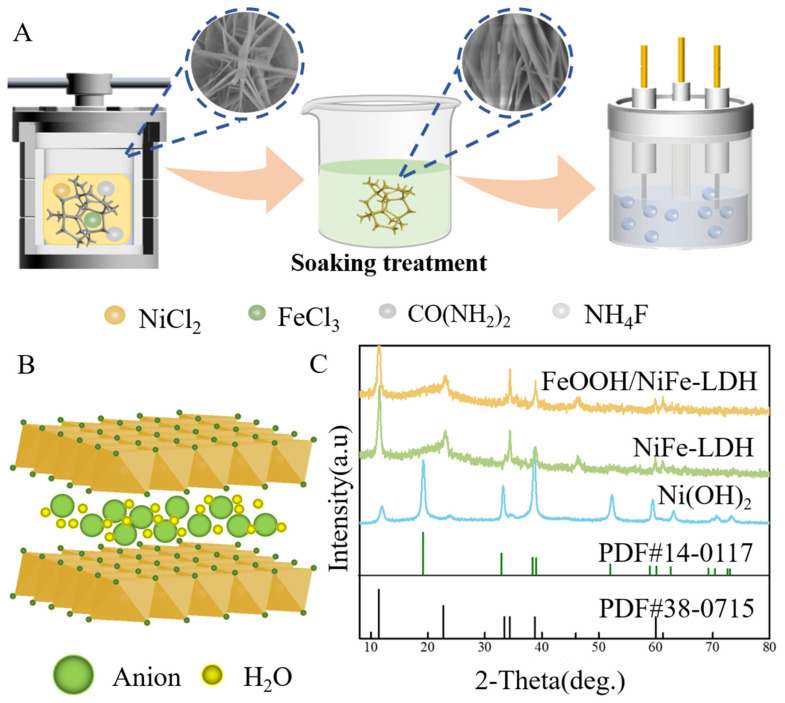
(**A**) Scheme of the preparation of FeOOH/NiFe-LDH; (**B**) representative crystal structures of LDH; (**C**) XRD spectrum of Ni(OH)_3_, Fe(OH)_3_, NiFe-LDH, and FeOOH/NiFe-LDH.

Figure 1A depicts a schematic representation of the synthesis procedure for depositing FeOOH/NiFe-LDH onto a nickel foam substrate and its operational mechanism during water electrolysis. The process begins with the synthesis of nanosheet-like NiFe-LDH on NF, achieved by modulating the nickel-to-iron ratio. This is followed by the development of FeOOH/NiFe-LDH through a solution impregnation process. The nanosheet-like NiFe bimetallic hydroxides provide an increased specific surface area, resulting in the exposure of larger active regions supporting the electrocatalytic reactions. For the solution immersion step, the micro-morphology of the sample evolves from the initially regular and smooth nanosheets to a rougher and fluffier nanosheet structure. This structural change reduces the interlayer gaps between the nanosheets, enabling improved electron transfer within the same spatial constraints. To confirm the structural composition of FeOOH/NiFe-LDH, XRD characterization was conducted on the samples (Figure 1B). The layered material consists of multiple positively charged layers interspersed with charge-balancing anions. These anions, situated between the layers, are relatively weakly bound and can typically undergo ion exchange. Within its layered structure, NiFe-LDH facilitates efficient electron transfer through metal cations and anions, which enhances its ability to catalyze OER. The crystallographic structures of the synthesized electrocatalysts were characterized by X-ray diffraction (Figure 1C). The pattern of Ni(OH)_2_ exhibits characteristic peaks at 19.3°, 33.1°, and 38.5° indexed to (001), (100), and (101) planes of hexagonal β-Ni(OH)_2_ (PDF#14-0117). For NiFe-LDH, distinct reflections at 11.2° (003), 22.8° (006), and 34.5° (012) confirm the hydrotalcite-type layered structure, consistent with typical NiFe-LDH phases [35,46]. The sharp and well-defined peaks observed stand for the excellent crystallinity of the NiFe-LDH and confirm the absence of any other heterogeneous phases. Following solution impregnation, the FeOOH/NiFe-LDH composite exhibits significantly attenuated diffraction peak intensities while retaining characteristic peak positions. This apparent amorphization suggests the attachment of a disordered FeOOH layer onto the crystalline NiFe-LDH nanosheet surfaces, with no observable impurity phases [47]. (The XRD spectrum of samples with different ratios is in Figure S4.)

**Figure 2:**
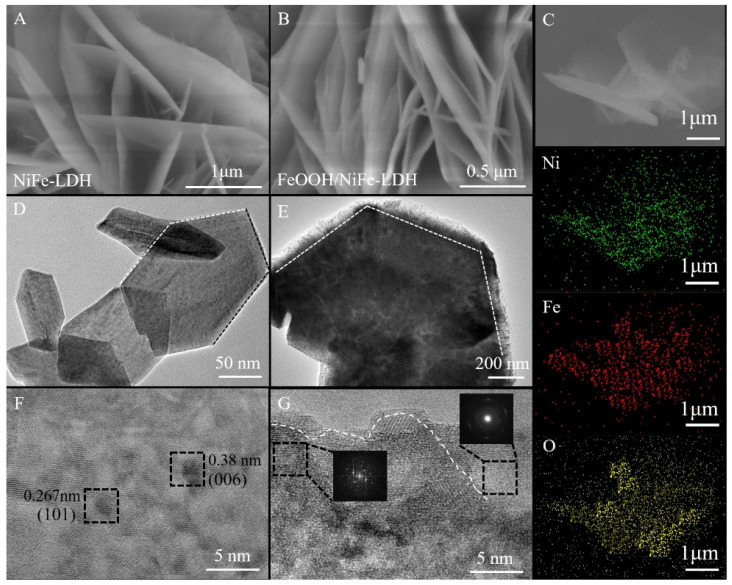
(**A**,**D**) HRTEM of NiFe-LDH; (**B**,**E**) HRTEM of FeOOH/NiFe-LDH; (**C**) corresponding EDS mapping images; (**F**,**G**) The lattice fringe of NiFe-LDH and FeOOH/NiFe-LDH, along with corresponding FFT transform.

SEM and TEM analyses were executed to obtain the microscopic appearance and structural characteristics of NiFe-LDH and FeOOH/NiFe-LDH. Figure 2 presents the corresponding SEM images, with additional SEM images of Ni(OH)_2_ and Fe(OH)_3_ provided in Figure S1 for reference. Figure 2A,D show the morphology of NiFe-LDH prior to the solution treatment. The nanosheets exhibit uniform thickness, smooth surfaces, and a well-defined porous structure formed by their interlaced stacking. Clear boundaries and gaps between the layers can be observed. After the solution treatment, significant morphological changes occur, as seen in Figure 2B,E. The originally compact nanosheets become delaminated, resulting in a fluffier and more disordered architecture with rougher surfaces. The edges of the nanosheets appear more irregular, suggesting surface reconstruction and possible amorphization. High-resolution TEM images in Figure 2F,G reveal the lattice fringes before and after treatment. In Figure 2F, distinct lattice fringes confirm the crystalline nature of pristine NiFe-LDH. In contrast, Figure 2G shows a largely disordered region with weak or indistinct lattice fringes, along with a selected area electron diffraction (SAED) pattern displaying broad, diffuse rings. These features indicate the formation of an amorphous or poorly crystalline FeOOH layer on the surface of the LDH nanosheets following treatment. Lastly, Figure 2C presents the EDS diagram after solution treatment. The pronounced and uniform elemental signals confirm a homogeneous distribution of elements across the material, with no evidence of phase heterogeneity.

**Figure 3:**
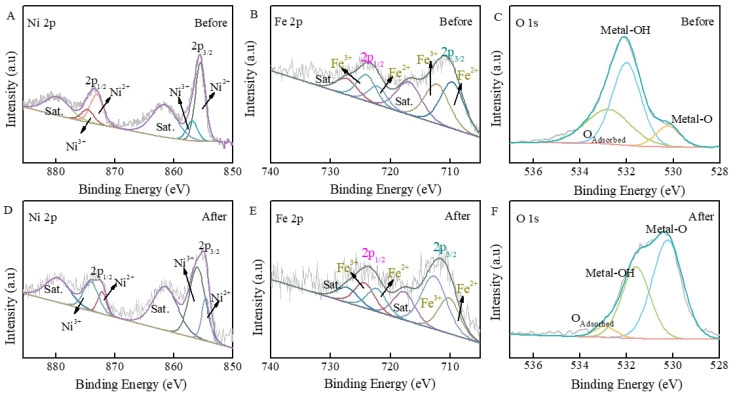
(**A**) XPS peaks before and after reaction; (**A**,**D**) Ni 2p; (**B**,**E**) Fe 2p; (**C**,**F**) O1s.

XPS serves as the characterization technique for assessing atomic binding energies and bond states through X-ray spectroscopy. Figure 3A illustrates the full spectrum of the FeOOH/NiFe-LDH sample, distinctly displaying the peaks corresponding to the Ni, Fe, and O elements. Figure 3B presents the high-resolution Ni 2p spectra, with two strong peaks at Ni 2p_3/2_ (855.4 eV, 856.8 eV) and Ni 2p_1/2_ (872.8 eV, 874.6 eV), which are accompanied by two satellite peaks indicative of the divalent and trivalent states of nickel ions, corresponding to Ni-O or γ-NiOOH. After the reaction, the peak area ratio of Ni^3+^ increased, which indicated that divalent Ni was oxidized to a trivalent state [9,48]. Figure 3C illustrates the high-resolution Fe 2p spectrum, which presents the peaks located at 710.2 eV, 712.8 eV, 722.1 eV, and 724.3 eV that correspond to Fe^2+^ 2p3/2, Fe^3+^ 2p3/2, Fe^2+^ 2p1/2, and Fe^3+^ 2p1/2. The 5.2 eV distance difference between the Fe-OH 2p_3/2_ peak (712.8 eV) and its satellite (718.0 eV) suggests the presence of a high-spin Fe^3+^ oxidation state. The binding energy shifts to the lower energy direction (about 0.7 eV), indicating that the coordination environment of Fe has changed [26,49]. In Figure 3D, the O1s spectrum can be deconvoluted into three distinct peaks (denoted O1, O2, O3), corresponding to lattice oxygen (M–O), surface hydroxyl groups (M–OH), and adsorbed oxygen species, respectively. The peak positions are observed at 530.3 eV (O1), 531.9 eV (O2), and 533.0 eV (O3) [45,46,47]. The O1 peak at 530.3 eV is attributed to the O1s electrons associated with the Metal-O bond, while the O2 and O3 peaks at 531.9 eV and 533 eV match with the Metal-OH bond and adsorbed oxygen, respectively [15,28], which further proves the surface defects and low coordination environment of FeOOH. The Fe 2p spectrum showed that the 2p_3/2_ peak of Fe^3+^ and the defective oxygen peak in the O 1s spectrum further confirmed the existence of surface defects.

### 3.2. HER Measurements

**Figure 4:**
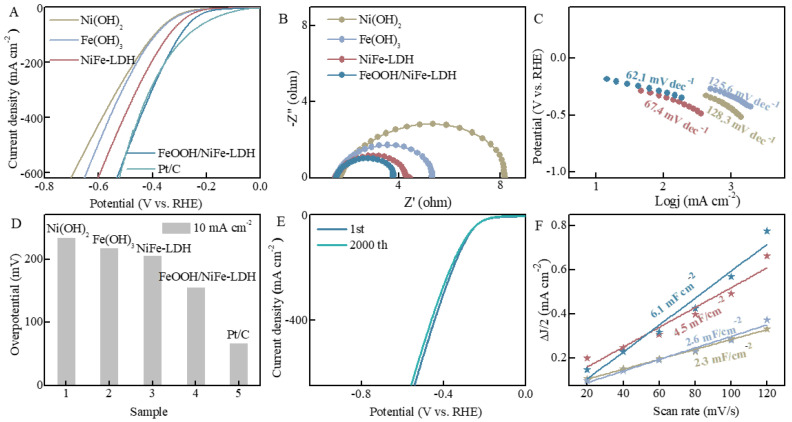
The HER performance of all the catalyst in 1M KOH. (**A**) LSV curves; (**B**) EIS diagrams; (**C**) Tafel plots; (**D**) overpotential; (**E**) LSV curves of the first and after the 2000th CV test; (**F**) corresponding double-layer capacitance.

The HER performance of FeOOH/NiFe-LDH and the control group samples was evaluated under the electrolyte of 1 M KOH, and the test parameter was consistent for all electrocatalysts. Before electrochemical testing, CV curve activation was performed to reach a steady state. By carefully analyzing the polarization curve, FeOOH/NiFe-LDH rapidly exceeds NiFe-LDH, Ni(OH)_2_, and Fe(OH)_3_ from the beginning of the reaction, and the current density continues to increase. Compared with the Pt/C electrode, the current density in the initial stage of the test is obviously lower. With the progress of the reaction, when the voltage is 0.35 V, the hydrogen production performance is almost comparable to that of Pt/C. The transfer ability of the charge is a crucial factor influencing the rate of HER/OER, which is depicted and assessed by the Nyquist diagram. As illustrated in Figure 4B, it is very intuitive from the figure that FeOOH/NiFe-LDH demonstrates the quickest charge-to-turn movement speed compared to NiFe-LDH, Ni(OH)_2_, and Fe(OH)_3_. The Tafel slopes for FeOOH/NiFe-LDH, NiFe-LDH, Ni(OH)_2_, and Fe(OH)_3_ were transformed by the LSV curve. Typically, a smaller Tafel slope indicates more rapid reaction kinetics and enhanced catalytic performance. In Figure 4C, FeOOH/NiFe-LDH, with the lowest Tafel slope value, is 62.1 mV dec^−1^, a value lower than NiFe-LDH (67.4 mV dec^−1^), Fe(OH)_3_ (125.6 mV dec^−1^), and Ni(OH)_2_ (128.3 mV dec^−1^), indicating that it exhibits the highest catalytic activity among the samples. As shown in Figure 4D, the HER overpotential of FeOOH/NiFe-LDH at current densities of 10 mA cm^−2^ is 155 mV, compared with NiFe-LDH (205 mV), Fe(OH)_3_ (217 mV), Ni(OH)_2_ (234 mV), and Pt/C (67 mV). The number of active sites is regarded as one of the criteria for assessing electrocatalytic performance. It can be compared by ECSA, and it tends to be acquired using the double-layer capacitance assay method. The CV curves of different catalysts at varying scanning velocities are displayed in Figure S9. In Figure 4F, the C_dl_ value of FeOOH/NiFe-LDH is 6.1 mF cm^−2^, which is better than NiFe-LDH (4.5 mF cm^−2^), Fe(OH)_3_ (2.6 mF cm^−2^), and Ni(OH)_2_ (2.3 mF cm^−2^). A higher ECSA value suggests that FeOOH offers a greater number of active regions during electrochemical reactions, thereby exhibiting superior catalytic efficiency. During the stability test of FeOOH/NiFe-LDH, it maintains good stability, with only minor variations after a long period of continuous testing.

### 3.3. OER Measurements

**Figure 5:**
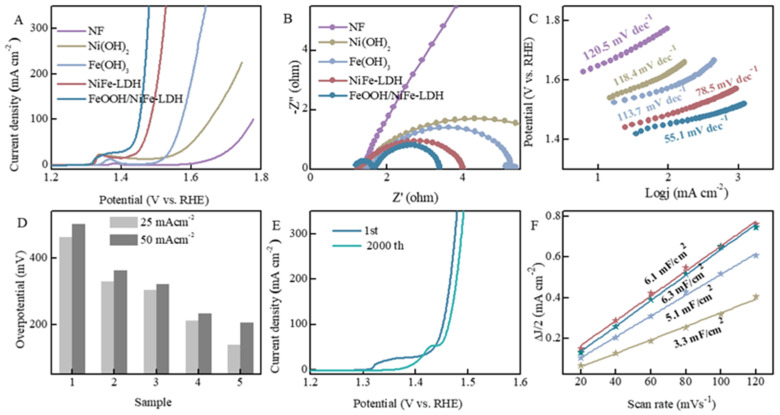
The OER performance of all the catalyst in 1M KOH. (**A**) LSV curves; (**B**) EIS diagrams; (**C**) Tafel plots; (**D**) overpotential; (**E**) LSV curves of the first and after the 2000th CV test; (**F**) corresponding double-layer capacitance.

The OER activity of FeOOH/NiFe-LDH and control group samples was evaluated usiing commercial RuO_2_ as a reference benchmark. After activation, the electrochemical characteristics were tested. Furthermore, the LSV demonstrates that the OER polarization curve of the sample exhibits a marked upward trend from the initial stage of the reaction with an increase in potential. In Figure 5A, the increase in the LSV curves of the NiFe-LDH compared with Ni(OH)_2_ and Fe(OH)_3_ represents a significant enhancement in OER activity. After further modification of the interface, its LSV curve also increases further, and FeOOH/NiFe-LDH shows the maximum current density at the same voltage. The overpotential at the standard current density (10 mA cm^−2^) is considered an important index for the preliminary evaluation of the electrocatalytic activity of OER. The overpotential of FeOOH/NiFe-LDH at the standard current density is 168 mV, and its current density consistently exceeds that of the other four groups of samples from the beginning of the reaction, so the control group requires higher additional power to drive the water reaction forward.

To thoroughly assess the kinetics of the OER performance of FeOOH/NiFe-LDH, Tafel slope analysis and EIS measurements were conducted. The doping of Fe ions adjusts the electronic structure, and the modification of FeOOH at the interface provided more active regions to further accelerate the reaction rate. Within the voltage range, the Tafel slope (55.1 mV dec^−1^) and impedance are notably the smallest, aligning with the anticipated results. This indicates that FeOOH/NiFe-LDH requires overcoming a relatively lower energy barrier and possesses minimal electron transfer impedance, thereby exhibiting exceptional electrocatalytic performance. The sequence of impedance values FeOOH/NiFe-LDH < NiFe-LDH < Fe(OH)_3_ < Ni(OH)_2_ is consistent with the LSV curve trend. Figure 5E presents the LSV curve after 2000 consecutive CV tests. The current density of the polarization curve remains high after a long period of continuous testing. The ECSA can be employed to approximately estimate the active sites (CV test curves with different sweeping speeds in Figure S10). ECSA is shown in Figure 5F, for which the C_dl_ value of FeOOH/NiFe-LDH is 6.3 mF cm^−2^, which is better than that of NiFe-LDH (6.1 mF cm^−2^), Fe(OH)_3_ (5.1 mF cm^−2^), and Ni(OH)_2_ (3.3 mF cm^−2^). The larger C_dl_ value indicates that FeOOH/NiFe-LDH can provide a larger active region, attributed to enhanced OER activity. Therefore, the differences in double-layer capacitance values strongly correlate with the observed variations in electrocatalytic activity and efficiency. NiFe-LDH modulates the electronic structure of the electrocatalyst, while the interfacial modification with FeOOH enhances the ECSA. These synergistic effects collectively contribute to improved electrocatalytic performance.

### 3.4. Hydrophilicity and Stability

**Figure 6:**
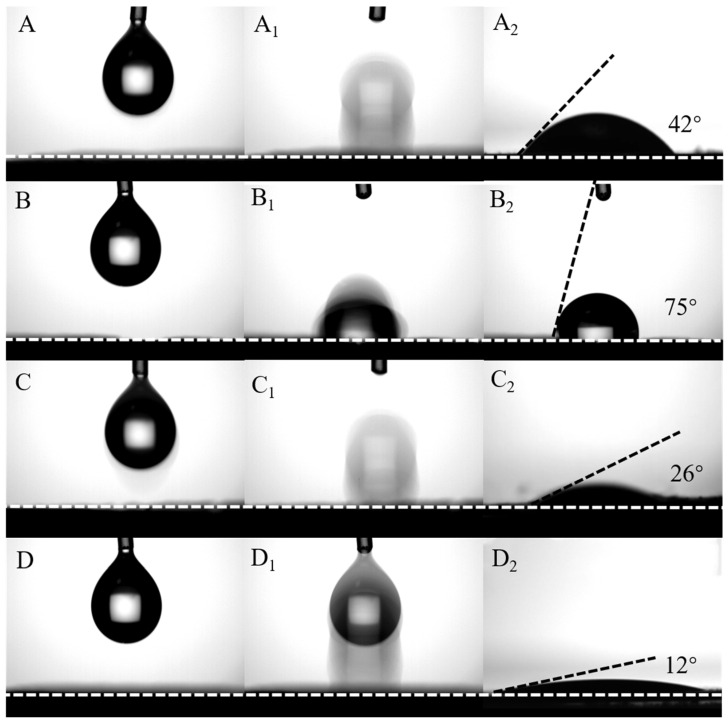
The contact angle measurements of (**A**,**A_1_**,**A_2_**) Ni(OH)_2_, (**B**,**B_1_**,**B_2_**) Fe(OH)_3_, (**C**,**C_1_**,**C_2_**) NiFe-LDH, and (**D**,**D_1_**,**D_2_**) FeOOH/NiFe-LDH.

The strong hydrophilicity of the electrocatalyst exterior can effectively minimize bubble adhesion, thus accelerating the desorption process of bubbles and increasing the exposure rate of active sites on the electrode exterior, thereby improving the performance. Based on the bubble mechanics model, its related equations are as follows:

(1) $$F_{b}+F_{d\;}={\;F}_{a}$$

(2) $$F_{b}=\frac{4}{3}\pi \gamma^{3}\Delta \rho g$$

(3) $$F_{d\;}=6\pi \mu \gamma v$$

(4) $$F_{a}=2\pi \gamma (1\;-\;\cos\theta )$$

where r represents the bubble radius; Δ*ρ* represents the variation in density between the liquid and gaseous states; g represents the acceleration of gravity; μ represents the hydrodynamic viscosity; v is the speed of movement of the bubble relative to the liquid; γ represents the gas–liquid interfacial tension; and θ represents the contact angle of the bubble and reflects the hydrophilicity of electrocatalysts. For the same electrocatalyst, in the process of electrolyzing water, assuming that the buoyancy force F_b_ and adhesion force F_d_ on the bubbles are constant, the smaller the adhesion force Fa, the easier the bubbles are released, and the faster the reaction rate. The stronger the hydrophilicity of the electrocatalyst, the smaller the contact angle θ, and cosθ approaches 1, thus significantly reducing the adhesion of F_a_. Shown in the inset of Figure 6D, the contact angle of the FeOOH/NiFe-LDH sample is 12°, where for NiFe-LDH it is 26° (Figure 6C–C_2_), Fe(OH)_3_ is 75° (Figure 6C–C_2_), and Ni(OH)_2_ is 42° (Figure 6B–B_2_), illustrating that FeOOH/NiFe-LDH has a more hydrophilic surface. The bubbles are subjected to less adhesion on the surface of the electrocatalyst, and the bubbles desorb faster.

**Figure 7:**
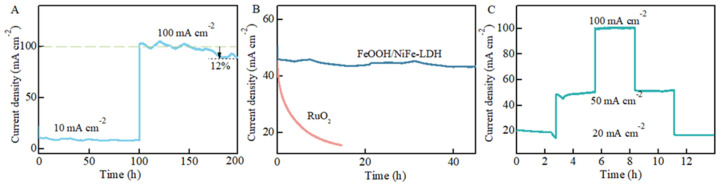
(**A**) I-T curve of FeOOH/NiFe-LDH at different current density; (**B**) I-T curves of FeOOH/NiFe-LDH and RuO_2_; (**C**) I-step stability test.

Stability is regarded as a pivotal parameter for assessing the suitability of electrocatalysts in industrial applications, particularly where sustained performance under high current densities is critical [9]. In Figure 7A, the I-T diagram demonstrates that FeOOH/NiFe-LDH has been subjected to sustained electrolysis at both high and low voltages. Despite the significant impact of the larger current density at high voltage on the electrode material, it consistently maintains a stable overpotential level. Notably, stability exhibits negligible variation after 200 h of testing. In addition, the continuous test of low voltage and high voltage within a fixed time range also shows a stable state in the multi-potential transition test (Figure 7B). In addition, Figure 7C gives the I-T curves of FeOOH/NiFe-LDH and RuO_2_. Therefore, the stability of FeOOH/NiFe-LDH is better than that of RuO_2_. After a long period of stability testing, there is a slight corrosion phenomenon on the surface of the sample. The sheet-like structure is weakly destroyed, as shown in Figure S2. To comprehensively analyze the catalytic performance of the FeOOH/NiFe-LDH electrocatalyst, a comparison with other electrocatalysts reported in the literature was conducted (Tables S1 and S2). The findings affirm that the FeOOH/NiFe-LDH catalyst demonstrates excellent stability and efficiency.

## 4. Conclusions

In this work, FeOOH/NiFe-LDH was successfully prepared by combining the hydrothermal method with solution impregnation. The synthesis route involved the growth of bimetallic Ni-Fe hydroxide nanosheets on NF, where the incorporation of Fe effectively modulated the intrinsic electronic structure and improved electrical conductivity. Subsequent interface modification with iron oxyhydroxide introduced significant structural changes through solution etching, creating numerous defects on the initially smooth nanosheet surfaces. This etching process also led to the attachment of irregular nanosheets at the interfaces, which further increased the number of active regions and facilitated electron transfer. The synergistic structural and electronic modifications are anticipated to significantly enhance both HER and OER activity. Electrochemical evaluation demonstrated that the synthesized FeOOH/NiFe-LDH nanosheets exhibited outstanding performance for the HER and OER, with overpotentials as low as 168 mV and 155 mV, respectively. Moreover, it displayed excellent long-term stability, suggesting its potential applicability in practical water splitting systems.

## Data Availability

Data are contained within the article and Supplementary Materials.

## Supplementary Materials

The following supporting information can be downloaded at: https://www.mdpi.com/article/10.3390/nano15120949/s1, Figure S1: (A,B) SEM of Ni(OH)_2_; (C,D) SEM of Fe(OH)_3_; Figure S2: (A,B) SEM images of FeOOH/NiFe-LDH after testing; Figure S3: (A,B) SEM images of NiFe-LDH and FeOOH/NiFe-LDH; (C,D) TEM images of NiFe-LDH and FeOOH/NiFe-LDH; Figure S4: The XRD spectrum of all the scale; Figure S5: The EIS of the initial and final test; Figure S6: The LSV of all the scale and different Fe ion treatment time; Figure S7: LSV curve after ECSA normalization; Figure S8: LSV curve after MA normalization; Figure S9: HER CV curves of Ni(OH)_2_, Fe(OH)_3_, NiFe-LDH, FeOOH/NiFe-LDH; Figure S10: OER CV curves of Ni(OH)_2_, Fe(OH)_3_, NiFe-LDH, FeOOH/NiFe-LDH; Figure S11: The LSV, EIS, Overpotential of FeOOH/NiFe-LDH and RuO_2_; Figure S12: The XPS full spectrum of FeOOH/NiFe-LDH before and after test; Table S1: Comparison of OER activity of the FeOOH/NiFe-LDH with other reported catalysts in alkaline electrolyte; Table S2: Comparison of HER activity of the FeOOH/NiFe-LDH with other reported catalysts in alkaline electrolyte. References [50,51,52,53,54,55,56,57] are cited in Supplementary Materials.
