# Supplementary material for: Rational Construction of Nano-Scaled FeOOH/NiFe-LDH for Efficient Water Splitting
Source: Nanomaterials (Basel). 2025 Jun 18;15(12):949. doi: 10.3390/nano15120949 (PMC12195736; doi:10.3390/nano15120949)
Supplement: Supplementary file 1 [file nanomaterials-15-00949-s001.zip › nanomaterials-3675255-supplementary.pdf]

Supporting Information

Rational Construction of Nano-Scaled FeOOH/NiFe-LDH for Efficient Water Splitting

Juan Yu [a], Xiubing Fu [a], Haoqi Wang [b,*], Shun Lu [c] and Bing Li [a,*]

a Anhui Province Key Laboratory of Pollutant Sensitive Materials and Environment Remediation, Anhui Province Industrial Generic Technology Research Center for Alumics Materials, School of Physics and Electronic Information, Huaibei Normal University, Huaibei 235000, China
b Radiation Technology Institute, Beijing Academy of Science and Technology, Beijing 100875, China
c Chongqing Institute of Green and Intelligent Technology, Chinese Academy of Sciences, Chongqing 400714, China
* Correspondence: Corresponding authors: wanghq@bnu.edu.cn (H.W.) and binglir@mail.ustc.edu.cn (B.L.)

Supporting Figures

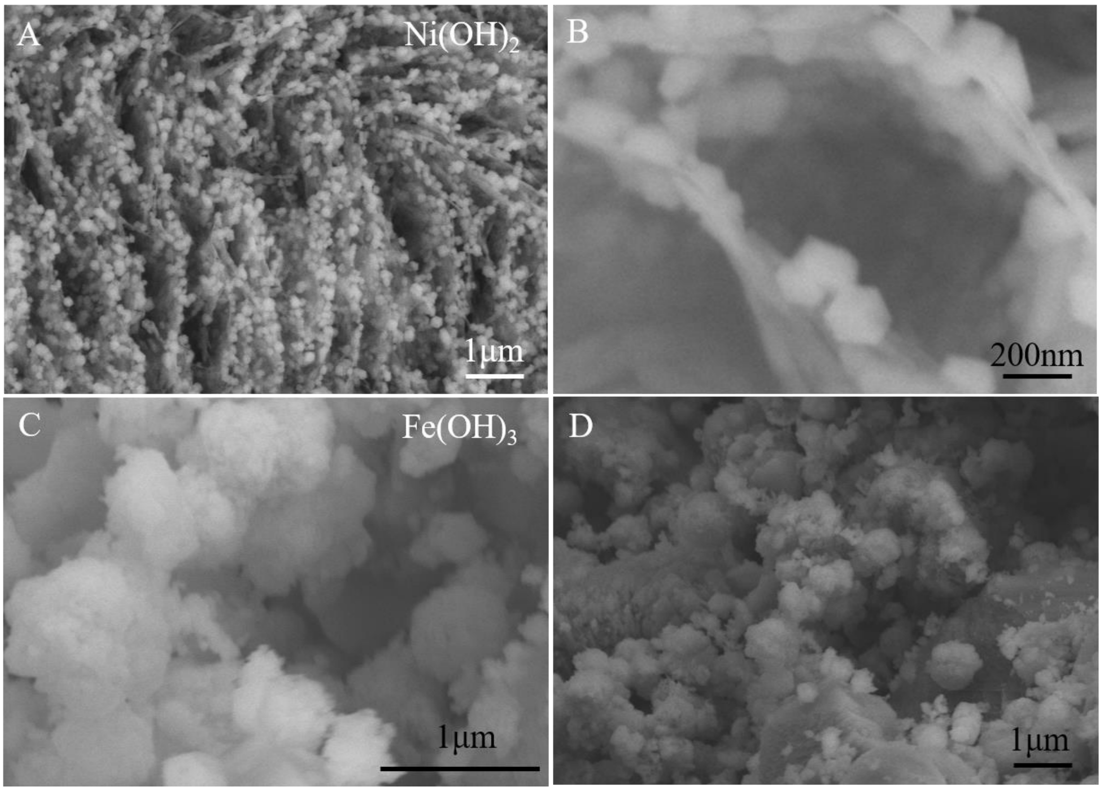

Figure S1. (A, B) SEM of $Ni(OH)_2$; (C,D) SEM of $Fe(OH)_3$.

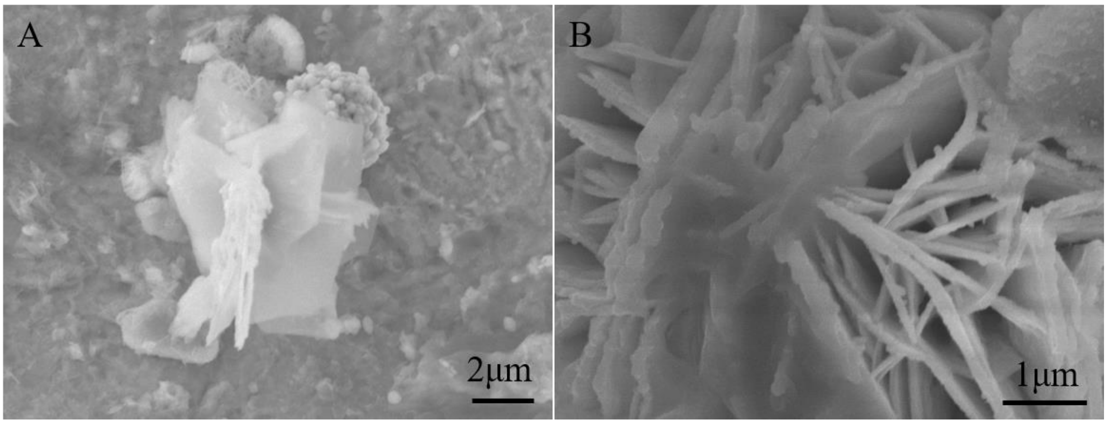

Figure S2. (A, B) SEM images of FeOOH/NiFe-LDH after testing.

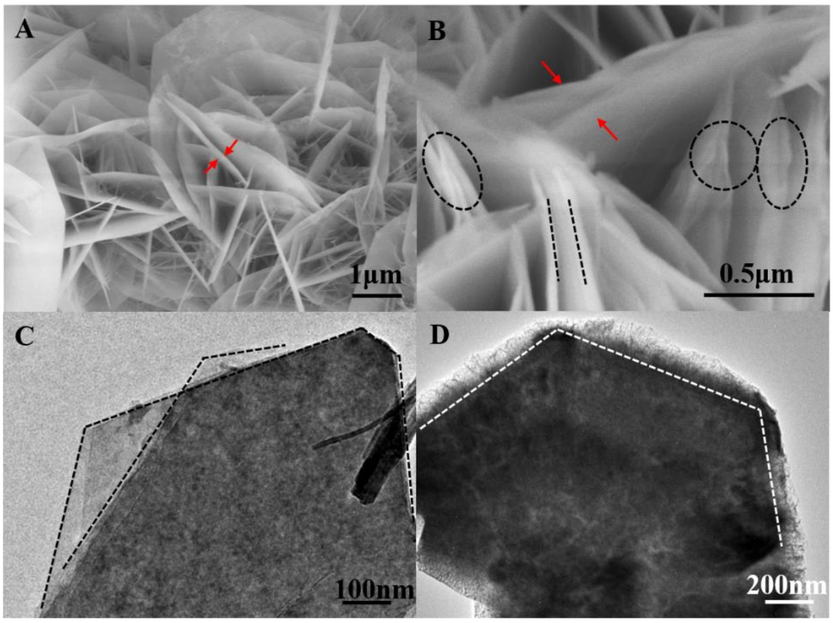

Figure S3. (A, B) SEM images of NiFe-LDH and FeOOH/NiFe-LDH; (C-D) TEM images of NiFe-LDH and FeOOH/NiFe-LDH.

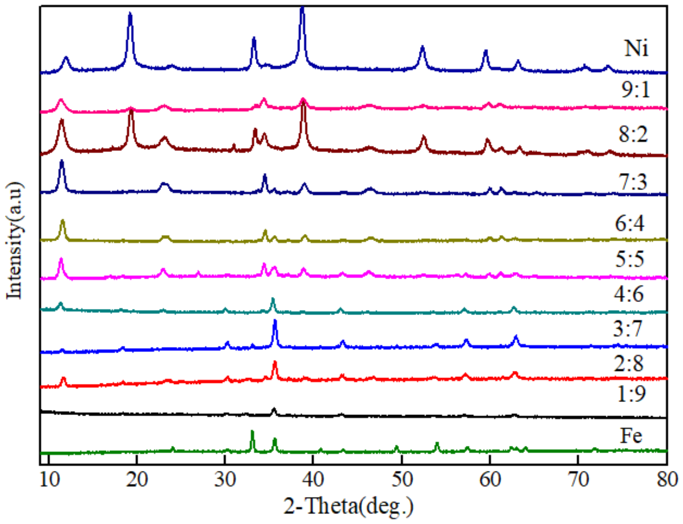

Figure S4. The XRD spectrum of all the scales.

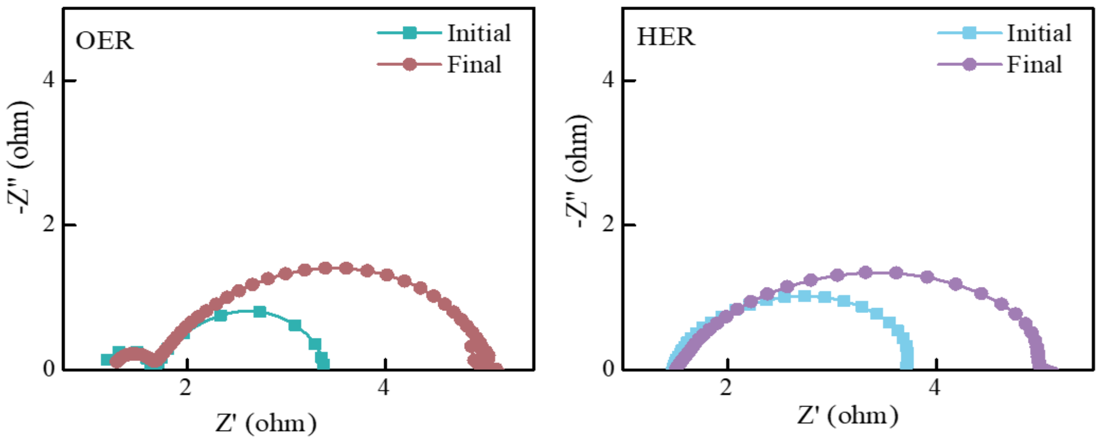

Figure S5. The EIS of the initial and final tests.

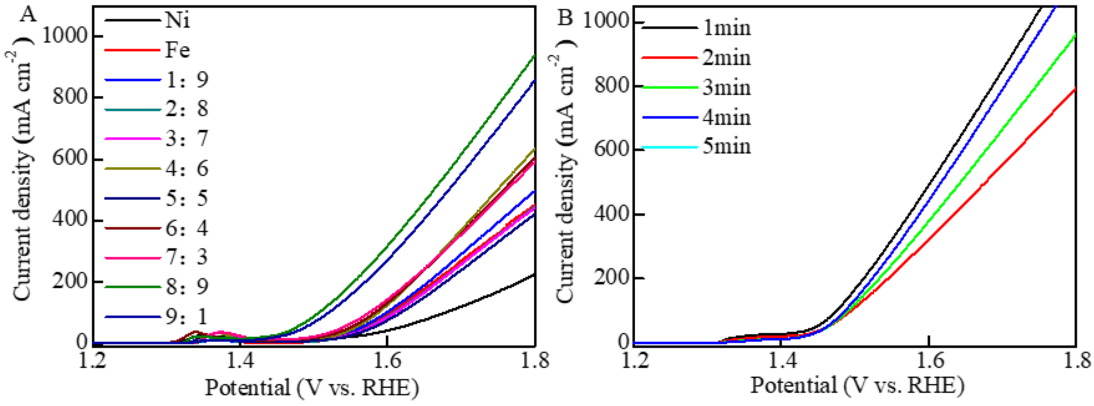

Figure S6. The LSV of all the scales and different Fe ion treatment times.

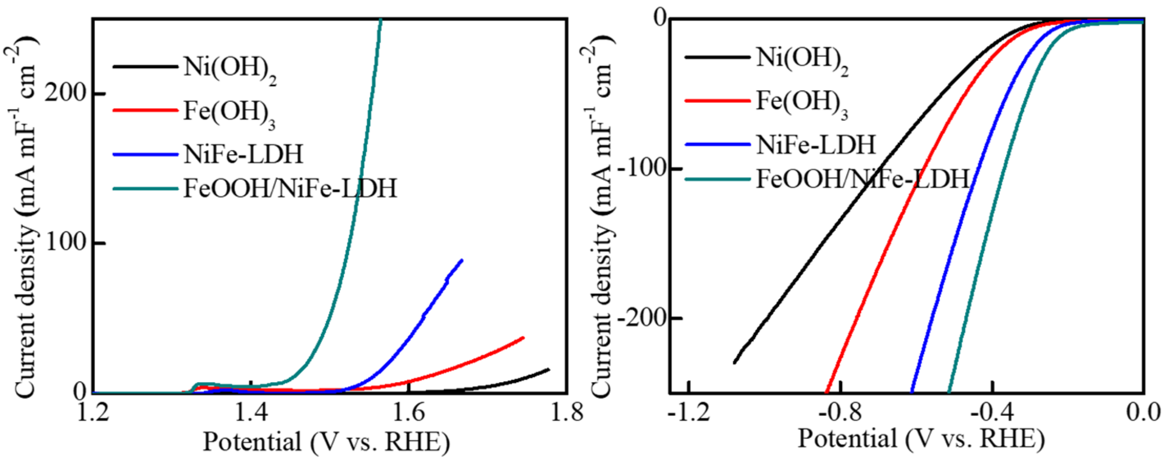

Figure S7. LSV curve after ECSA normalization.

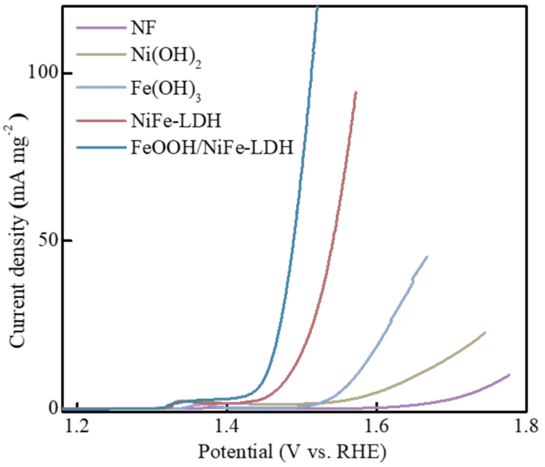

Figure S8. LSV curve after MA normalization.

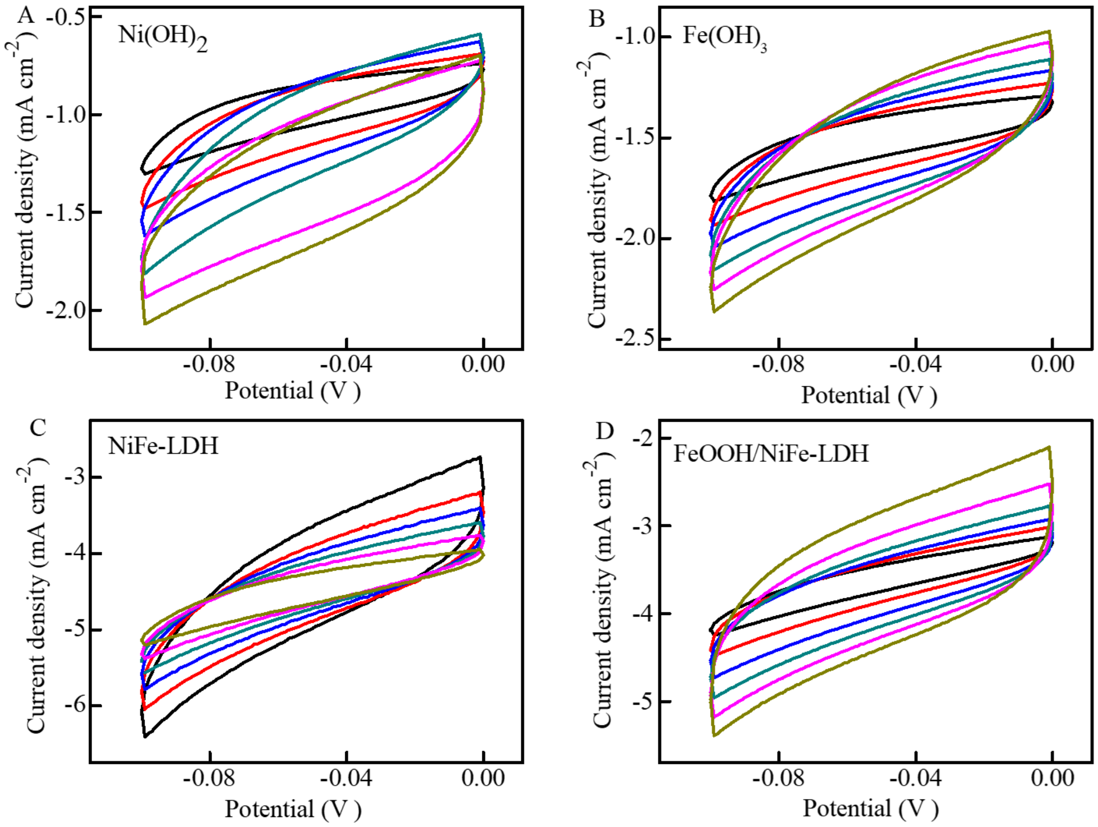

Figure S9. HER CV curves of $Ni(OH)_2$, $Fe(OH)_3$, NiFe-LDH, and FeOOH/NiFe-LDH.

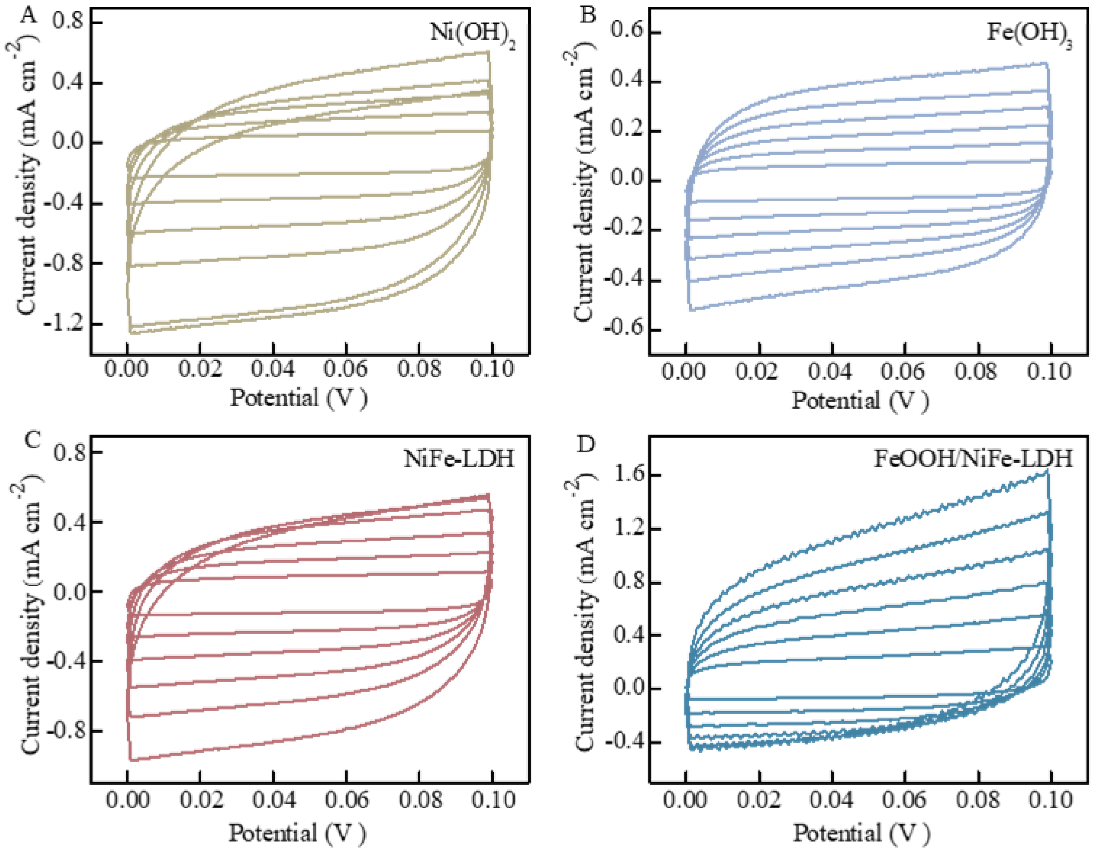

Figure S10. OER CV curves of $Ni(OH)_2$, $Fe(OH)_3$, NiFe-LDH, and FeOOH/NiFe-LDH.

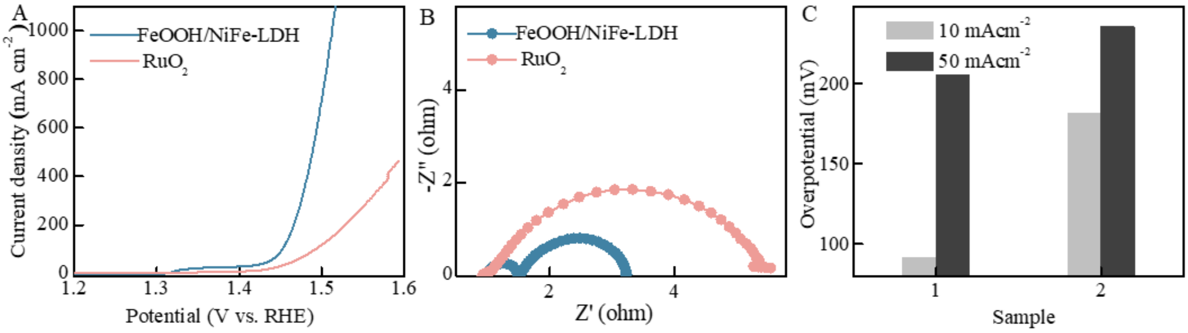

Figure S11. The LSV, EIS, and overpotential of FeOOH/NiFe-LDH and RuO_2.

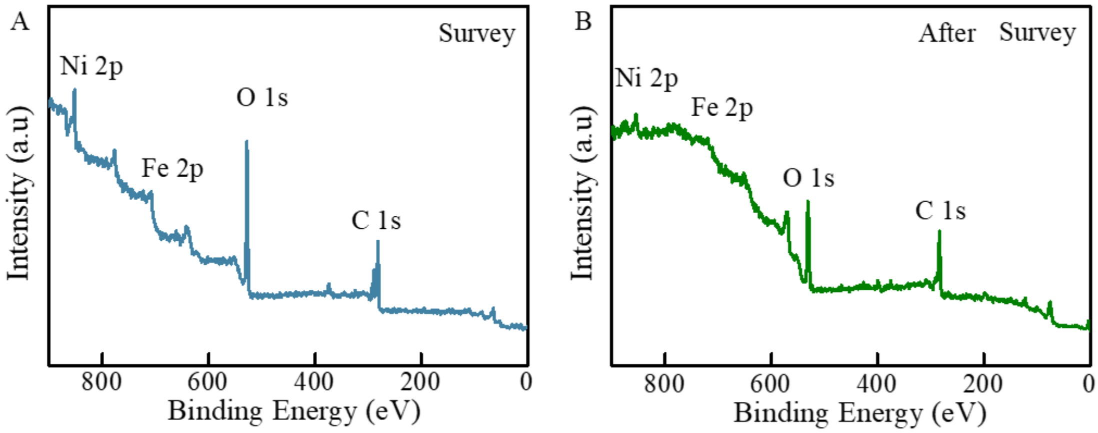

Figure S12. The XPS full spectrum of FeOOH/NiFe-LDH before and after the test.

Table S1. Comparison of OER activity of the FeOOH/NiFe-LDH with the other reported catalysts in alkaline electrolyte.

Electrocatalysts	Overpotential(mV) at 10 mA cm^{-2}	Stability (h)	Tafel slop (mV dec^{-1})	Ref.
FeOOH/NiFe-LDH	91	200	55.1	This work
Co-Ni_3S_2	154	25	75	[4]
Fe_3O_4/Au/CoFe-LDH	89	2	--	[50]
NiTe@FeOOH	179(at 100 mA cm^{-2})	100	52	[51]
FeOOH-Co_9S_8	215(at 100 mA cm^{-2})	24	46.6	[31]
FeCo-LDH/PANI	153	35	45	[52]
Ni-Co-Se	260	95	--	[53]
$NiCo_2S_4$/FeOOH	200	20	73	[38]

Table S2. Comparison of HER activity of the FeOOH/NiFe-LDH with the other reported catalysts in alkaline electrolyte.

Electrocatalysts	Overpotential (mV)	Stability (h)	Tafel slop (mV dec^{-1})	Ref.
FeOOH/NiFe-LDH	155	--	62.1	This work
Fe-Co-P	87	28	63	[54]
Ru-CuO-SA	12.9	50	27.9	[55]
CoFe@NiFe-200/NF	240	30	88	[23]
$Co_{81}Ni_{19}$	132	10	84.3	[56]
Fe-Co-CN/rGO-700	215	45	54	[57]
